# Flagellin-induced NADPH oxidase 4 activation is involved in atherosclerosis

**DOI:** 10.1038/srep25437

**Published:** 2016-05-05

**Authors:** Jinoh Kim, Misun Seo, Su Kyung Kim, Yun Soo Bae

**Affiliations:** 1 Department of Life Science, Ewha Womans University, Seoul, Korea

## Abstract

It is widely accepted that bacterial infection-mediated inflammation facilitates development of atherosclerosis by activating toll-like receptor (TLR) signaling system. We reasoned that NADPH oxidases (Nox), required for TLR-mediated inflammatory response, are involved in atherogenesis. Here, we show that the activation of Nox4 through TLR5 regulates the inflammation of the endothelium and in atherogenesis. Flagellin-induced interaction between the COOH region of Nox4 and the TIR domain of TLR5 led to H_2_O_2_ generation, which in turn promoted the secretion of pro-inflammatory cytokines including IL-8, as well as the expression of ICAM-1 in human aortic endothelial cells (HAECs). Knockdown of the Nox4 in HAECs resulted in attenuated expressions of IL-8 and ICAM-1 leading to a reduction in the adhesion and trans-endothelial migration of monocytes. Challenge of recombinant FliC (rFliC) to the ApoE KO mice with high-fat diet (HFD) resulted in significantly increased atherosclerotic plaque sizes compared to the saline-injected mice. However, an injection of rFliC into the Nox4ApoE DKO mice with HFDs failed to generate atherosclerotic plaque, suggesting that Nox4 deficiency resulted in significant protections against rFliC-mediated atherogenesis. We conclude that TLR5-dependent Nox4 activation and subsequent H_2_O_2_ generation play critical roles for the development of atherosclerosis.

It is well known that toll-like receptors (TLRs) specifically recognize pathogen-associated molecular patterns (PAMPs), including lipopolysaccharide (LPS), bacterial lipoprotein, bacterial CpG DNA, and flagellin, and then they induce innate immune responses, resulting in expressions of various pro-inflammatory cytokines^1^. It has been well established that atherosclerosis is mediated by chronic inflammation in the blood vessels^2^. Atherosclerosis involves the accumulation of lipids in the arterial intima, leading to the induced formation of atherosclerotic lesions, which are mediated by inflammation, cell death and fibrosis.

It has been reported that bacterial infection is associated with the pathogenesis of atherosclerosis. Many epidemiological studies have suggested that a large number of pathogens, including *Chlamydia pneumonia*, *Helicobacter pylori*, *Porphyromonas gingivalis*, and *Actinobacillus actinomycetemcomitans*, are associated with the elevated risk of atherosclerosis^3^,^4^,^5^. Periodontitis is known to be associated with atherosclerosis and it is a good example for explaining the link between bacterial infection and atherosclerosis^6^,^7^. Although the molecular mechanism that the pathogens can accelerate atherogenesis needs to be clarified, the activation of TLR is crucial to the initiation of an inflammatory response to atherosclerosis^8^,^9^. In human atherosclerotic lesions, expressions of TLR2, TLR4, and TLR5 are up-regulated when compared to a healthy artery^10^. Therefore, vascular cells are sensitive to the ligands of various expressions of TLR2, TLR4, and TLR5, leading to the development of atheroma^11^. Several reports have concentrated on TLR2- and TLR4-mediated atherosclerosis^8^,^9^,^12^. However, the molecular link between TLR5 as a bacterial flagellin receptor and atherosclerosis is still unclear.

Flagellin is a globular protein that assembles the filament in the bacterial flagellum. It has been reported that flagellin binds to TLR5 and activates TLR5 downstream signaling cascades, such as the IRAK-TRAF6 complex and nuclear factor-kB (NF-kB), leading to induced inflammatory responses^1^,^13^. NADPH oxidase (Nox) is known to serve functional roles in TLR-mediated innate immune responses. We previously reported that Duox2 plays a critical role in flagellin-induced inflammatory responses, including IL-8 production and the MUC5AC expression in the airway epithelia^14^. It has been demonstrated that Nox is a key player in TLR-mediated intracellular signaling cascades^14^,^15^,^16^,^17^. The Nox family consists of Nox1-5 and Duox1-2. Among them, the Nox1, Nox2, and Nox4 isozymes are expressed in the cardiovascular tissues. The function of the Nox isozymes is not only to regulate normal vascular physiology but also to contribute to the development of cardiovascular disease including atherosclerosis^18^,^19^. We elucidated that LPS activates Nox4 through a direct interaction of TLR4, leading to reactive oxygen species (ROS) generation and NFkB activation. This pathway promotes the secretion of chemokines such as interleukin 8 (IL-8) and monocyte chemotactic protein 1 (MCP-1) and the expression of an adhesion molecule, such as intercellular adhesion molecule 1 (ICAM-1), providing a pro-inflammatory condition in human aortic endothelial cells (HAECs)^16^. Moreover, we reported that the stimulation of the smooth muscle cells (SMCs) with Pam3CSK4 as an agonist TLR2 induces ROS generation through the activation of Nox1^17^. This pathway induces the activation of matrix metalloprotease 2 (MMP-2) and the secretion of macrophage inflammatory protein 2 (MIP-2), leading to the migration of the smooth muscle cells (SMCs)^17^.

Even though vascular cells are responsive to the ligands of TLR2, TLR4, TLR5, and TLR9 during the development of carotid atheroma^11^, many studies have been primarily focused on the effects of TLR2 and TLR4 on atherosclerosis. Here, we show a molecular link between TLR5 and atherosclerosis through the activation of the Nox isozyme. We demonstrate that flagellin as a TLR5 agonist promoted H_2_O_2_ generation through Nox4 activation and then induced the expression of ICAM-1 on the surfaces of endothelial cells and the secretion of IL-8, resulting in endothelial inflammation. We verified the effect of Nox4 on atherogenesis in Nox4-deficient ApoE knockout mice. These results demonstrate that the Nox4 isozyme plays an important role in TLR5-mediated H_2_O_2_ generation, which in turn stimulates inflammation in the endothelial cells and the development of atherosclerosis.

## Results

### TLR5 interacts with Nox1 and Nox4

**Figure 1:**
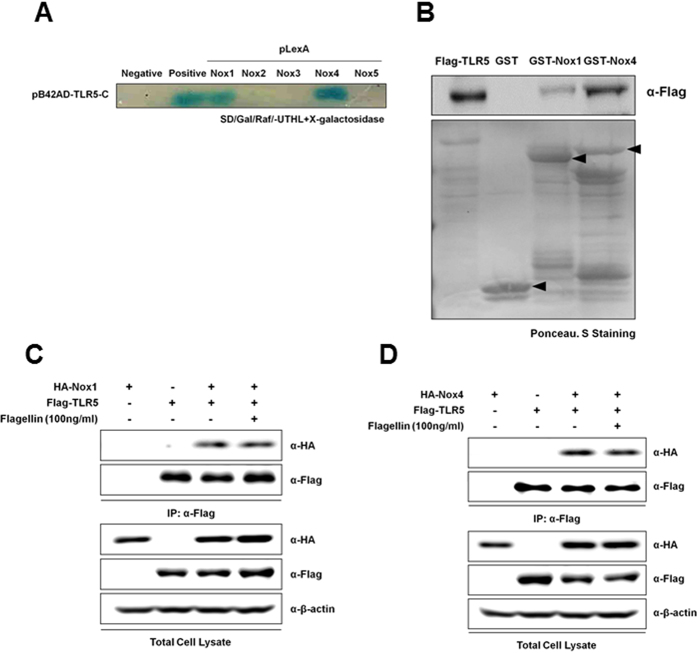
TLR5 interacts with Nox1 and Nox4. (**A**) The direct interaction of the TLR5 with Nox1–5 C was estimated by yeast-two hybrid assay. Following the selection for Trp^+^ and His^+^ phenotype, Leu dependent growth and β-galactosidase activity were tested in an incubation medium (SD/galactose/Raffinose). (**B**) GST alone, GST-Nox1 (AA 217–550) and Nox4 (AA 248–578) were tested for their interactions with HEK293T cell lysates expressing Flag-tagged TLR5, and Flag-tagged TLR5 proteins were detected using an anti-Flag. Flag-CMV-TLR5 was transiently expressed in HEK293T cells either alone or together with pcDNA3.0-HA-Nox1C (AA 217–550) (**C**) or pcDNA3.0-HA-Nox4C (AA 250–573) (**D**), as indicated. After serum starvation, flagellin (100 ng/ml) was treated for 30 min. Cell lysates were then subjected to immunoprecipitation (IP) with antibodies to Flag, and the resulting precipitates were subjected to immunoblot analysis with antibodies to HA.

To identify which Nox isozymes can interact with TLR5, we performed yeast two-hybrid assay using Toll/Interleukin-1 receptor (TIR, AA 691–837) domain of TLR5 as a bait and the COOH-terminal region of various human Nox isozymes. Yeast cells expressing pB42AD- TIR-TLR5 with pLexA-Nox1-C (COOH-terminal region, AA 217–550) and pLexA-Nox4-C (COOH-terminal region, AA 354–578) revealed normal growth, and they produced blue colonies in the absence of leucine and in the presence of X-gal, indicating that the TIR region of TLR5 interacts with COOH-terminal region of Nox1 as well as that of Nox4 (Fig. 1A). However, other Nox isozymes (Nox2, AA 291–571; Nox3, AA 296–568; Nox5, AA 395–720) failed to interact with TLR5 (Fig. 1A). To confirm the yeast two-hybrid screening results, we next performed GST pull-down assay and co-immunoprecipitation. GST fusion proteins containing the COOH-terminal regions of Nox1 or Nox4 were conjugated with glutathione-Sepharose 4B beads, and then the bead-conjugated GST fusion proteins were incubated with lysates of HEK293T cells expressing the TLR5 (Flag-CMV-TLR5). We found that the TLR5 interacted with the COOH-terminal region of Nox1 or of Nox4 (Fig. 1B). Co-immunoprecipitation assay was performed to identify whether TLR5 binds with Nox1 or Nox4 in response to flagellin (Fig. 1C,D). The HEK293T cells were transfected with Flag-CMV-TLR5 and HA-Nox1-C or HA-Nox4-C and incubated in the absence or presence of flagellin. Cell lysates were immunoprecipitated with antibody to Flag. An immunoblot analysis of the resulting immune complex revealed that TLR5 interacted with both Nox1-C and Nox4-C. However, the extent of these interactions was not affected by flagellin stimulation, suggesting that TLR5 constitutively interacts with Nox1 or Nox4 in a flagellin-independent manner.

### Nox4 is required for flagellin-induced ROS generation

**Figure 2:**
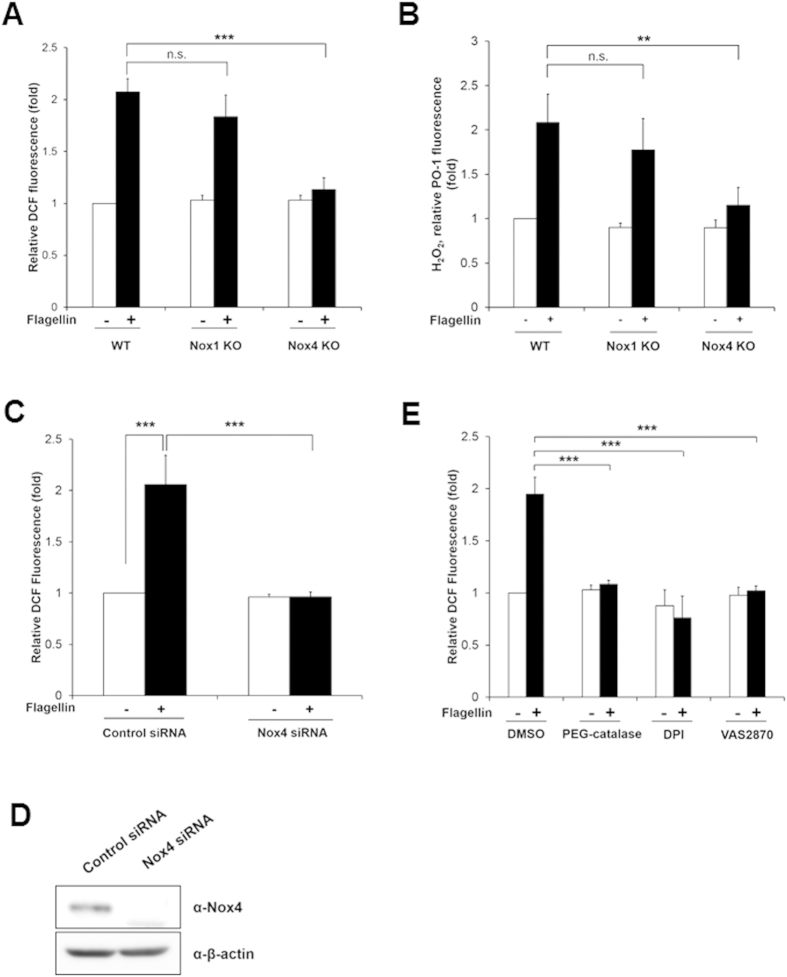
Nox4 is required for flagellin-induced ROS generation. (**A**) Wild type (WT), Nox1 knockout (KO), and Nox4 KO MAECs were stimulated for 10 min with flagellin (100 ng/ml), H_2_O_2_ was monitored by a confocal microscopic analysis of DCF fluorescence (N = 3, mean ± SD, ***p < 0.001). (**B**) WT, Nox1 KO, and Nox4 KO MAECs were incubated for 10min with flagellin (100 ng/ml), H_2_O_2_ was monitored by a confocal microscopic analysis of PO-1 fluorescence (N = 3, mean ± SD, **p < 0.01). (**C**) HAECs were transiently transfected with control or Nox4 siRNA, and then incubated for 10 min with flagellin (100 ng/ml), H_2_O_2_ was monitored by a confocal microscopic analysis of DCF fluorescence (N = 3, mean ± SD. ***p < 0.001). (**D**) Nox4 protein expression was analyzed by immunoblotting with an antibody against Nox4. (**E**) HAECs were pretreated with PEG-catalase (50 U/ml) or DPI (20 μM) or VAS2870 (5 μM) for 30 min, and stimulated for 10 min with flagellin (100 ng/ml), H_2_O_2_ was monitored by a confocal microscopic analysis of DCF fluorescence (N = 3, mean ± SD, ***p < 0.001).

To verify the effect of Nox1 or Nox4 on the ROS generation by flagellin, we prepared primary mouse aortic endothelial cells (MAECs) from wild type (WT), Nox1 knockout (KO), or Nox4 KO mice (Supplementary Figure S1). The flagellin stimulated ROS generation in the MAECs from wild type (WT) or Nox1 KO mice (Fig. 2A). However, the MAECs from the Nox4 KO mice failed to induce ROS generation in response to flagellin stimulation (Fig. 2A). The result strongly indicated that flagellin-mediated ROS generation is achieved with Nox4, not Nox1, even if TLR5 interacts with both isozymes. To identify the ROS species in MAECs in response to flagellin, we used Peroxy Orange-1 (PO-1) dye which is specifically sensitive to H_2_O_2_^20^. Increasing PO-1 fluorescence as an indication of H_2_O_2_ generation was detected in MAECs from wild type and Nox1 KO in response to flagellin, whereas PO-1 fluorescence was significantly reduced in MAECs from Nox4 KO (Fig. 2B). The result strongly indicated that the predominant species of ROS induced by flagellin in MAECs is H_2_O_2_.

Next, we tested whether flagellin mediates H_2_O_2_ generation in human aortic endothelial cells (HAECs). HAECs were transfected with siRNA specific to Nox4, and the knockdown efficiency of Nox4 was analyzed by immunoblot assay. The immunoblot analysis with an antibody to Nox4 indicated that the Nox4 expression was abolished in HAECs transfected with Nox4 siRNA (Fig. 2D). HAECs transfected with Nox4 siRNA failed to induce H_2_O_2_ generation in response to flagellin stimulation, compared with the cells transfected with control siRNA (Fig. 2C). These results indicated that the activation of Nox4 is essential for flagellin-induced H_2_O_2_ generation in vascular cells. We investigated the effect of polyethylene glycol-catalase (PEG-catalase) as a H_2_O_2_ scavenger, diphenyleneiodonium (DPI) as a broad oxidase inhibitor, or VAS2870 as a Nox-specific inhibitor on flagellin-mediated H_2_O_2_ generation in HAECs. Pre-incubation of HAECs with PEG-catalase, DPI, or VAS2870 resulted in inhibition of H_2_O_2_ generation in response to flagellin (Fig. 2E).

### TLR5-Nox4 complex is involved in flagellin-induced H_2_O_2_ generation and NF-kB activation in HAECs

**Figure 3:**
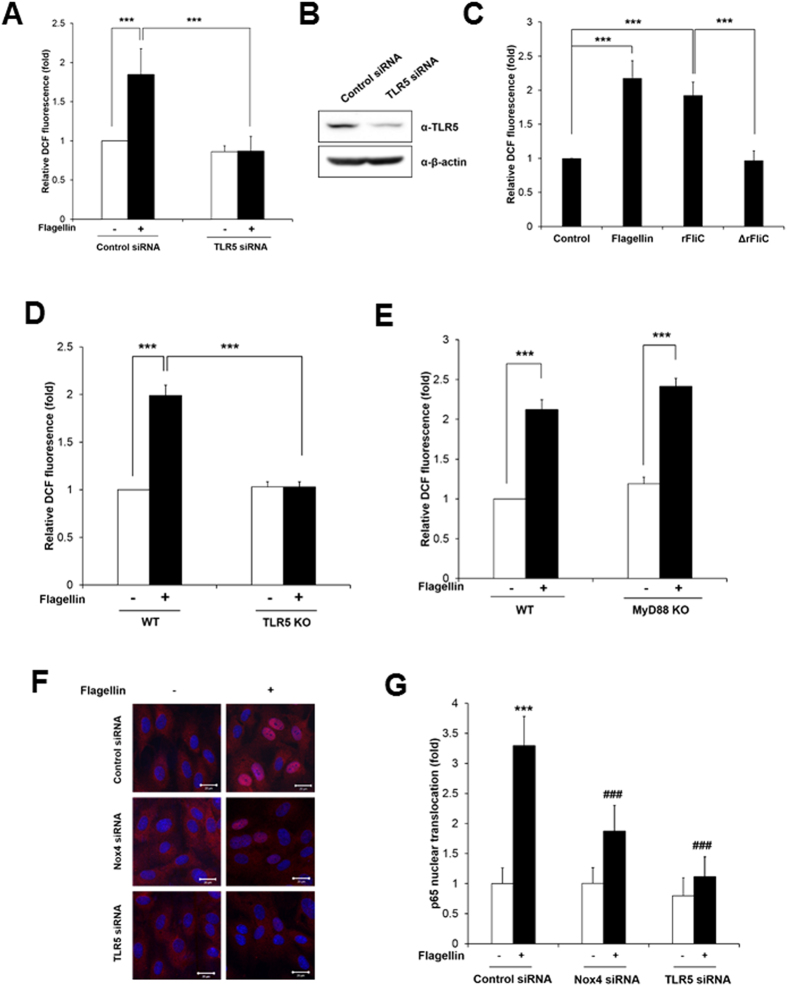
Nox4 is required for flagellin-induced NF-kB activation in HAECs. (**A**) HAECs were transfected with control or TLR5 siRNA, and stimulated for 10 min with flagellin (100 ng/ml). H_2_O_2_ was monitored by a confocal microscopic analysis of DCF fluorescence (N = 3, mean ± SD, ***p < 0.001). (**B**) TLR5 protein expression was analyzed by immunoblotting with an antibody against TLR5. (**C**) HAECs were stimulated with flagellin (100 ng/ml), rFliC (100 ng/ml), or ΔrFliC (100 ng/ml) for 10 min. H_2_O_2_ was monitored by confocal microscopic analysis of DCF fluorescence. (N = 3, mean ± SD, ***p < 0.001). (**D**) Wild type and TLR5 knockout MAECs were stimulated for 10 min with flagellin (100 ng/ml). H_2_O_2_ was monitored by confocal microscopic analysis of DCF fluorescence. (N = 3, mean ± SD, ***p < 0.001). (**E**) Wild type and MyD88 knockout MAECs were stimulated for 10 min with flagellin (100 ng/ml). H_2_O_2_ was monitored by confocal microscopic analysis of DCF fluorescence (N = 3, mean ± SD, ***p < 0.001). (**F**) HAECs were transfected with control siRNA (non-targeting siRNA), Nox4 siRNA, or TLR5 siRNA, stimulated with flagellin (100 ng/ml) for 1 hr and stained with antibody to p65 (red) and DAPI (blue). A representative image is shown. Scale bar: 20 μm. (**G**) Nuclear translocated p65 was quantified using an image J program (N = 3, mean ± SD, ***Comparison with non-stimulated control group, p < 0.001, ^###^Comparison with flagellin stimulated control group, p < 0.001).

To confirm the involvement of TLR5 as a bacterial flagellin receptor in such an event, we explored the effect of TLR5 knockdown on flagellin-induced H_2_O_2_ generation in HAECs. TLR5 protein expression was abolished in HAECs transfected with TLR5 siRNA (Fig. 3B). The HAECs transfected with TLR5 siRNA failed to induce H_2_O_2_ generation in response to flagellin, compared with the cells transfected with control siRNA (Fig. 3A). We examined the effect of additional TLR4 or TLR2 ligand on ROS generation in HAEC transfected siRNA specific to TLR5. HAECs transfected with TLR5 siRNA stimulated to induce H_2_O_2_ generation in response to LPS as a TLR4 ligand or Pam3CSK4 as a TLR2 agonist (Supplementary Figure S2). These results indicate that the activation of TLR5 is a crucial event in flagellin-mediated H_2_O_2_ generation. Moreover, we examined whether the complex of TLR5 with flagellin is involved in H_2_O_2_ generation. We prepared *Salmonella typhimurium* recombinant FliC (rFliC, AA21–505, purity 92.5%, Supplementary Figure S3) as an active component for triggering TLR5 and N-terminal region-deleted mutant recombinant FliC (ΔrFliC; AA119–505, purity 91.4%, Supplementary Figure S3) which cannot interact with TLR5^13^. The stimulation of HAECs with the rFliC protein resulted in increased H_2_O_2_ production, whereas the mutant rFliC protein (ΔrFliC) failed to induce H_2_O_2_ generation in the HAECs (Fig. 3C). We next prepared primary mouse aortic endothelial cells (MAECs) from TLR5 KO mice. Stimulation of primary MAECs from the TLR5 KO mice with flagellin failed to induce ROS generation in response to flagellin stimulation (Fig. 3D). These results indicated that the TLR5-flagellin complex plays an important role in H_2_O_2_ generation.

It has been reported that the activation of TLR5 by flagellin mediated the recruitment of the cytoplasmic adaptor proteins MyD88 and TIRAP/Mal, followed by the formation of a complex with IRAKs and TRAF6 that eventually led to the activation and nuclear translocation of NF-kB^1^. As MyD88 plays a pivotal role in TLR5-mediated signaling, we examined the function of MyD88 in flagellin-mediated H_2_O_2_ generation. We prepared MAECs from MyD88 KO mice and measured H_2_O_2_ generation in response to flagellin. Flagellin stimulated H_2_O_2_ generation in MAECs from MyD88 KO as well as WT mice, suggesting that flagellin-induced H_2_O_2_ generation is mediated by a MyD88-independent pathway (Fig. 3E). Next, we tested whether TLR5-induced H_2_O_2_ generation induces the activation of NF-kB, which is a hallmark of inflammatory signaling. The stimulation of HAECs with flagellin resulted in the increased nuclear localization of RelA/p65 as a subunit of NF-kB (Fig. 3F). However, the silencing of Nox4 or TLR5 in the HAECs showed a significantly reduced nuclear localization of RelA/p65, indicating that a flagellin-induced TLR5-Nox4 cascade stimulates NF-kB signaling (Fig. 3G).

### TLR5-Nox4-NF-kB signaling cascade regulates flagellin-induced pro-inflammatory mediator production in HAECs

**Figure 4:**
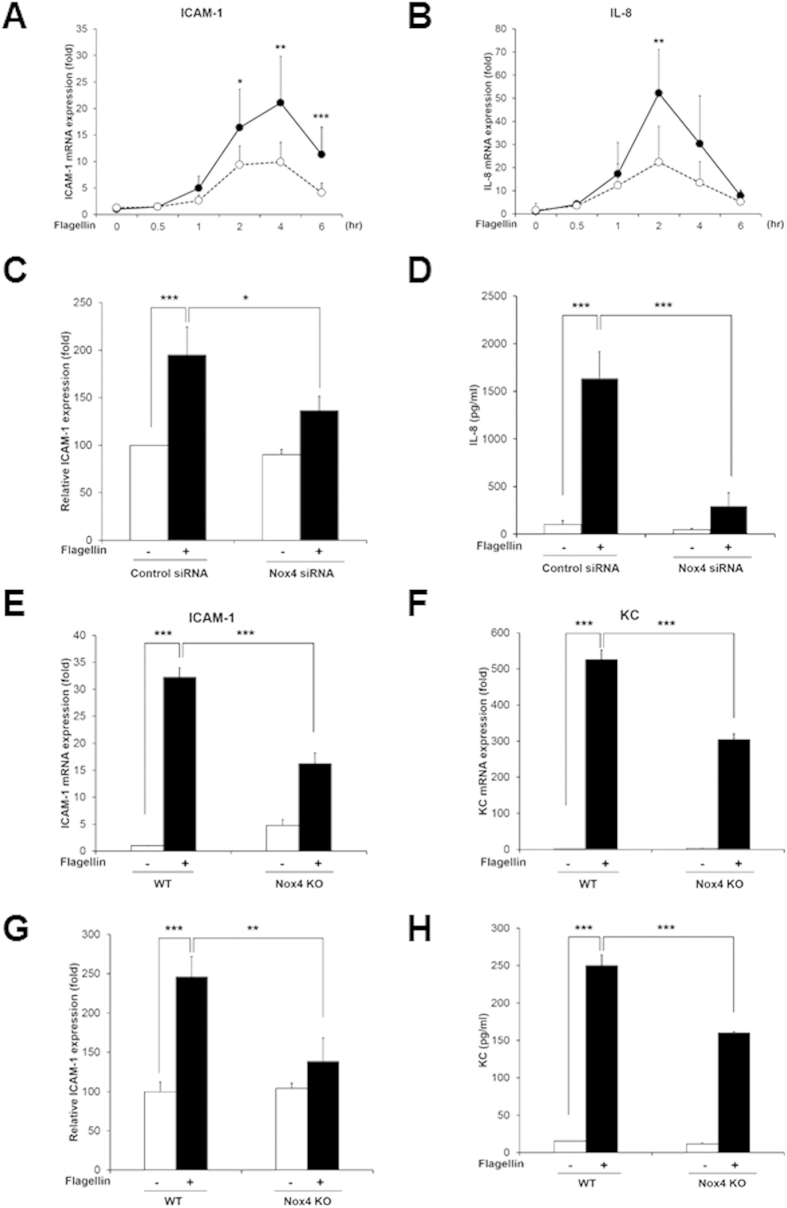
Nox4 regulates flagellin-induced ICAM-1 and IL-8 production. (**A**) Quantification of ICAM-1 mRNA expression in HAECs (Comparison with Nox4 siRNA group, *p < 0.05, **p < 0.01 and ***p < 0.001, black circle; control siRNA, white circle; Nox4 siRNA). (**B**) Quantification of IL-8 mRNA expression in HAECs (Comparison with Nox4 siRNA group, **p < 0.01, black circle; control siRNA, white circle; Nox4 siRNA). (**C**) HAECs were stimulated with flagellin (100 ng/ml) for 16 hrs. The ICAM-1 protein expression level was determined by a flow cytometric analysis, and 10,000 cells were routinely analyzed in FACScalibur (N = 3, mean ± SD, *p < 0.05, ***p < 0.001). (**D**) HAECs were stimulated with flagellin (100 ng/ml) for 12 hrs. The IL-8 protein secretion was analyzed by ELISA (N = 3, mean ± SD, ***p < 0.001). MAECs from WT and Nox4 KO were stimulated with flagellin (100 ng/ml) for 2 hrs (**E**,**F**). (**E**) Quantification of ICAM-1 mRNA expression in MAECs (N = 5, mean ± SD, ***p < 0.001). (**F**) Quantification of KC mRNA expression in MAECs (N = 5, mean ± SD, ***p < 0.001). (**G**) MAECs from WT and Nox4 KO mice were stimulated with flagellin (100 ng/ml) for 16 hrs. The ICAM-1 protein expression level was determined by a flow cytometric analysis, and 10,000 cells were routinely analyzed in FACScalibur (N = 3, mean ± SD, **p < 0.01, ***p < 0.001). (**H**) MAECs from WT and Nox4 KO mice were stimulated with flagellin (100 ng/ml) for 12 hrs. The KC protein secretion was analyzed by ELISA (N = 3, mean ± SD, ***p < 0.001).

To test the notion that the activation of the flagellin-TLR5-Nox4 cascade stimulates NF-kB as a key transcription factor in the inflammation of endothelial cells, we first explored whether flagellin-induced H_2_O_2_ generation mediates the expressions of various pro-inflammatory markers. The expressions of intercellular adhesion molecule-1 (ICAM-1) and interleukin-8 (IL-8) were induced by flagellin (Fig. 4A,B). However, knockdown of Nox4 in HAECs resulted in significantly reduced expressions of ICAM-1 and IL-8 in response to flagellin (Fig. 4A,B). We investigated the ICAM-1 expression on the surfaces of HAECs, which are analyzed with an antibody to ICAM-1 in the FACS analysis. The ICAM-1 expression on the surfaces of HAECs transfected with Nox4 siRNA in response to flagellin was significantly reduced, compared with that of HAECs transfected with control siRNA (Fig. 4C). Moreover, we measured the protein level of IL-8 secretions using an enzyme-linked immunosorbent assay (ELISA). The stimulation of HAECs with flagellin increased the secretion of IL-8 protein, while the silencing of Nox4 in HAECs remarkably decreased it (Fig. 4D). These results suggest that flagellin stimulates the expression of a pro-inflammatory mediator, such as IL-8 or ICAM-1, through the Nox4-NF-kB cascade.

**Figure 5:**
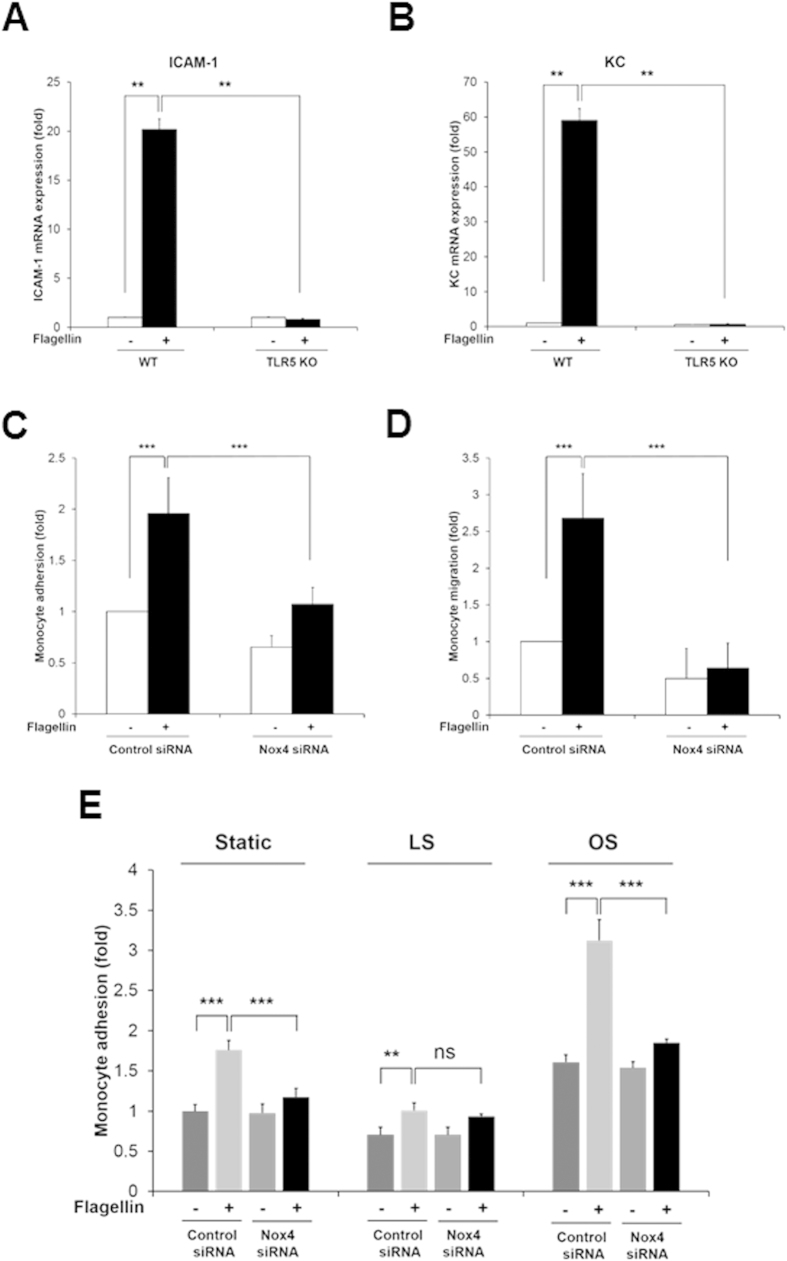
TLR5-deficient MAECs fails to induce the expression of pro-inflammatory molecules and Nox4 regulates monocyte adhesion and migration in response to flagellin. MAECs from WT and TLR5 KO were stimulated with flagellin (100 ng/ml) for 2hrs (**A**,**B**). (**A**) Quantification of flagellin-dependent ICAM-1 mRNA expression in MAECs from WT or TLR5 KO mice (N = 3, mean ± SD, **p < 0.01). (**B**) Quantification of flagellin-induced KC mRNA expression in MAECs from WT or TLR5 KO mice (N = 3, mean ± SD, **p < 0.01). (**C**) Control or Nox4 siRNA-transfected HAECs were stimulated in the absence or presence of flagellin (100 ng/ml). Pre-stained U937 cells with BCECF-AM (5 μg/ml) were incubated with HAECs for 30 min. The number of adherent U937 cells was determined (N = 3, mean ± SD, ***p < 0.001). (**D**) HAECs were transfected control or Nox4 siRNA in lower chamber and then stimulated with flagellin. U937 cells in upper chamber were allowed to migrate on fibronectin-coated Transwell plates for 4 hr. Trans-migrated U937 cells were counted under an inverted fluorescence microscope (N = 3, mean ± SD, ***p < 0.001). (**E**) Monocyte adhesion assay in flow condition. HAECs transfected control siRNA or Nox4 siRNA were exposed to laminar shear stress (15 dyn/cm^2^) or oscillatory shear stress (±5 dyn/cm^2^) with or without flagellin (100 ng/ml) for 24 hrs using the cone-and-plate apparatus. Pre-stained U937 cells with BCECF-AM (5 μg/ml) were incubated with HAECs for 30 min. The number of adherent U937 cells was determined (N = 3, mean ± SD, **p < 0.01, ***p < 0.001).

To validate the effect of Nox4 on expressions of ICAM-1 and mouse keratinocyte-derived chemokine (KC) as a mouse homolog of IL-8 in response to flagellin, primary MAECs from Nox4 KO mice were prepared. Stimulation of Nox4-deficient MAECs with flagellin resulted in attenuated mRNA and protein level of ICAM-1 and KC expression, compared with wild type MAECs (Fig. 4E–H). Moreover, we examined pro-inflammatory cytokines in primary MAECs from the TLR5 KO mice. Incubation of TLR5 KO MAECs with flagellin revealed suppressed expression of ICAM-1 and KC (Fig. 5A,B). These results clearly show that flagellin-TLR5-Nox4 cascade is essential for the production of pro-inflammatory cytokines including ICAM-1 and IL-8.

To verify the effect of Nox on expression of ICAM-1 and IL-8, HAECs was pretreated with diphenylene iodonium (DPI) as a Nox inhibitor or VAS2870 as a Nox-specific inhibitor and then analyzed for the expression of these molecules in HAECs. Indeed, pre-treatment of HAECs with DPI or VAS2870 resulted in significantly decreased expression of ICAM-1 and IL-8 (Supplementary Figure S4). To determine the function of TLR5 on the expression of these inflammatory molecules, we used N-terminal region-deleted mutant recombinant FliC (ΔrFliC; AA119–505, purity 91.4%, Supplementary Figure S3). Stimulation of HAEC cells with ΔrFliC failed to induce the expression of ICAM-1 and IL-8 (Supplementary Figure S5). Moreover, the expressions of these molecules were significantly abrogated upon pretreatment with Bay11–7084 as a NF-kB inhibitor (Supplementary Figure S6), suggesting that flagellin-induced H_2_O_2_ generation stimulates the expressions of ICAM-1 and IL-8 through TLR5-Nox4-NF-kB signaling cascade.

### Nox4 regulates flagellin-induced monocyte adhesion on endothelial cells and trans-endothelial migration

Monocyte-endothelial cell interactions are thought to be critical for the initiation and progression of atherosclerosis^21^. ICAM-1 contributes to the interaction between leukocytes and endothelial cells and IL-8 serves as a chemoattractant for recruiting monocytes and for inducing monocyte adhesion to the vascular endothelium^22^. Therefore, we investigated the effect of Nox4 activity on flagellin-mediated monocyte adhesion in endothelial cells. HAECs were stimulated with flagellin for 6 h and then adhesion of U937 cells to HAECs was stained with BCECF-AM. The flagellin-induced adhesion of monocytic U937 cells to HAECs was significantly inhibited by the downregulation of Nox4 in HAECs transfected with Nox4 siRNA, compared with the HAECs transfected with control siRNA (Fig. 5C). To evaluate the function of Nox4 in the trans-endothelial migration of monocytes, HAECs were plated in the bottom chamber of transwells, and monocytic U937 cells were added to the top chamber for 4 h. The number of U937 cells that migrated to the flagellin-activated endothelial cells in the bottom chamber was determined. The flagellin-induced migration of monocytic U937 cells was remarkably reduced by the silencing of Nox4 in the HAECs transfected with Nox4 siRNA, compared with the control cells transfected with control siRNA (Fig. 5D). These results demonstrated that the activation of Nox4 by flagellin is essential for the interaction between monocytes and endothelial cells.

It has been well established that disturbed flow in branched arteries induces inflammatory diseases such as atherosclerosis^23^,^24^. We investigated the effect of flow condition (laminar shear stress, LS and oscillatory shear stress, OS) on cell adhesion assay in HAEC transfected with control siRNA or Nox4 siRNA. Exposing HAEC to LS flow (HAEC-LS) for 24 h in cone-and-plate device induced regular cell shape with flow direction and stimulated KLF2 expression as a marker protein for LS flow (Supplementary Figure S7)^25^. However, OS flow (HAEC-OS) for 24 h disrupted regular shape leading to cobblestone shape formation and induced VCAM-1 expression as an atherosclerosis-prone protein (Supplementary Figure S7)^26^. Incubation of HAEC-OS with U937 cell resulted in significantly increased U937 adhesion in response to flagellin stimulation, compared to HAEC-LS (Fig. 5E). Silencing of Nox4 in HAEC-OS led to inhibit U937 adhesion in response to flagellin (Fig. 5E). The result indicated that Nox4 plays an important role in oscillatory shear stress-induced adhesion of monocytes.

### Nox4-deficient mice lead to the protection against rFliC-mediated atherosclerosis

**Figure 6:**
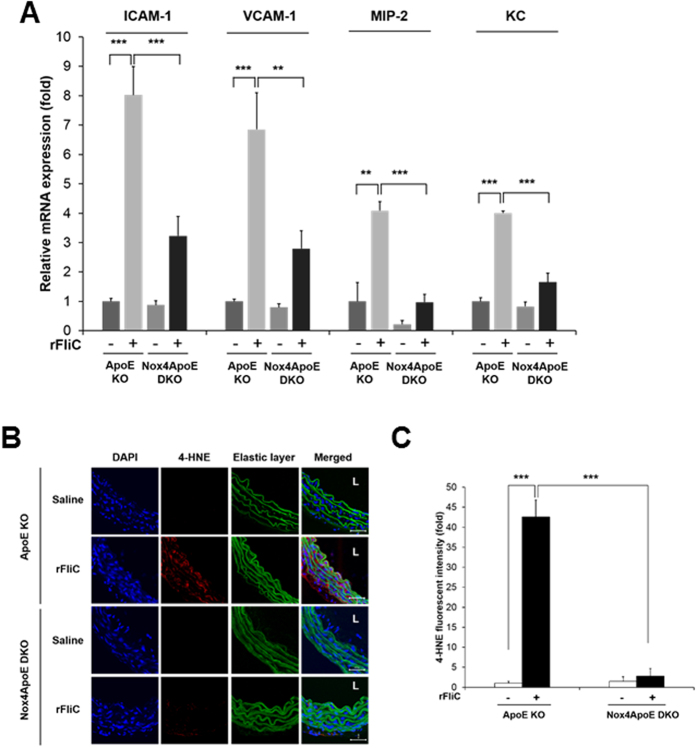
Nox4 regulates ROS generation and flagellin-induced pro-inflammatory mediator production in ApoE knockout mice. (**A**) Expression of a pro-inflammatory mediator was assayed by real-time PCR using endothelial enriched RNA obtained from the thoracic aorta after 2 hrs of i.v. injection with saline or rFliC (25 μg/mouse). Endothelial cell purity was qualified in each RNA (SMA/PECAM-1 <0.001). Quantification of the ICAM-1 mRNA expression, ***p < 0.001. Quantification of the VCAM-1 mRNA expression, **p < 0.01, ***p < 0.001. Quantification of the MIP-2 mRNA expression, **p < 0.01, ***p < 0.001. Quantification of KC mRNA expression, ***p < 0.001. (N = 3, mean ± SEM). (**B**) Thoracic aorta sections of ApoE KO and Nox4ApoE DKO mice were stained with 4-hydroxy-2-nonenal (4-HNE) for detection of oxidative stress-dependent lipid peroxidation. L means lumen. Scale bar: 50 μm. (**C**) Quantification of fluorescence intensity of 4-HNE staining using image J. (N = 3, mean ± SEM, ***p < 0.001).

We investigated whether activation of Nox4 can regulate the production of a flagellin-induced pro-inflammatory mediator in the aortic endothelial cells using ApoE knockout (KO) or Nox4ApoE double knockout (DKO) mice. We isolated the aortas from the ApoE KO and Nox4ApoE DKO mice with tail-vein injections of rFliC or saline, and mRNA from the endothelial cells were obtained (see Methods)^27^. The purities of the endothelial cells were measured by determining the ratio of SMA/PECAM-1. The ratios under 0.001 suggested that the mRNA was from mouse aortic endothelial cells (MAECs) and not from aortic SMCs or leukocytes. The expression of various pro-inflammatory mediators, including ICAM-1, VCAM-1, and the functional IL-8 homologues such as MIP-2 and KC^28^ was induced in MAECs from rFliC-injected ApoE KO mice. However, the expressions of these mediators were abrogated in MAECs from rFliC-injected Nox4ApoE DKO mice (Fig. 6A). These results clearly indicated that Nox4 mediates flagellin-induced ICAM-1, VCAM-1, MIP-2 and KC gene expressions in MAECs, leading to the development of atherosclerosis.

It has been well established that 4-hydroxy-2-nonenal (4-HNE) is known to be produced by oxidative stress in animal tissues^29^. The 4-HNE as a product of Nox4-dependent H_2_O_2_ generation was determined by immunohistochemistry with antibody against 4-HNE. Production of 4-HNE in thoracic aorta from rFliC-injected ApoE KO mice was significantly increased compared with that from saline-injected ApoE KO mice (Fig. 6B,C). However, 4-HNE production dramatically reduced in the aorta of rFliC-injected Nox4ApoE DKO mice (Fig. 6B,C). This result demonstrated that production of 4-HNE was mediated by Nox4-induced H_2_O_2_ generation in the aorta of mice.

**Figure 7:**
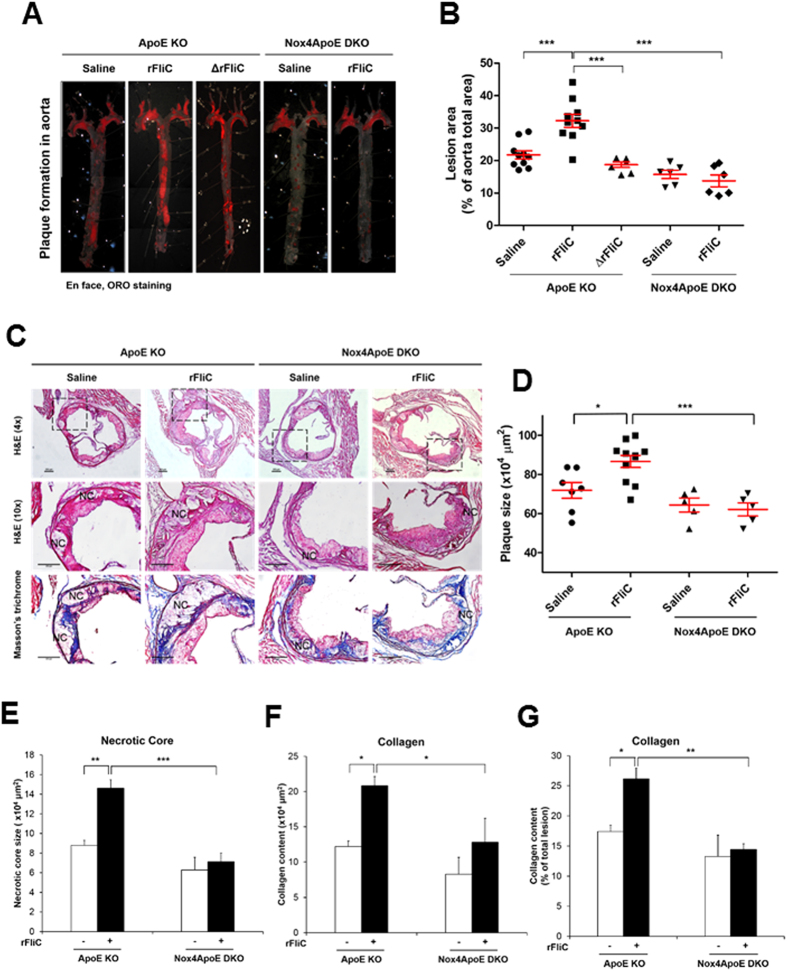
Nox4ApoE DKO mice lead to the protection against rFliC-mediated atherosclerosis. (**A**) ApoE KO mice and Nox4ApoE DKO mice were fed with HFDs for 12 weeks and were injected intravenously every week with 25 μg of rFliC, ΔrFliC or saline. The aortas were dissected, Oil-Red-O stained, and pinned. (**B**) The percent of the *en face* aortic surface that contained lesions was measured using Image J. (N = 5 to 10, mean ± SEM. ***p < 0.001). (**C**) Sinus sections were obtained from ApoE KO or Nox4ApoE DKO mice fed high-fat diet for 12 weeks with or without rFliC injection. H&E staining (top 2 images) and masson’s trichrome staining (bottom image). Collagen contents (blue) and necrotic core size (i.e. area with almost complete loss of collagen) were measured using image J software. Scale bar = 250 μm. (**D**) Quantitative analysis of the plaque size, N = 5 to 10, mean ± SEM, *p < 0.05, ***p < 0.001 (**E**) Quantitative analysis of necrotic core size, N = 5 to 10, mean ± SEM, **p < 0.01, ***p < 0.001 (**F**) Quantitative analysis of collagen area, N = 5 to 10, mean ± SEM, *p < 0.05. (**G**) Quantification of collagen content in total lesion area, N = 5 to 10, mean ± SEM, *p < 0.05, **p < 0.01.

**Figure 8:**
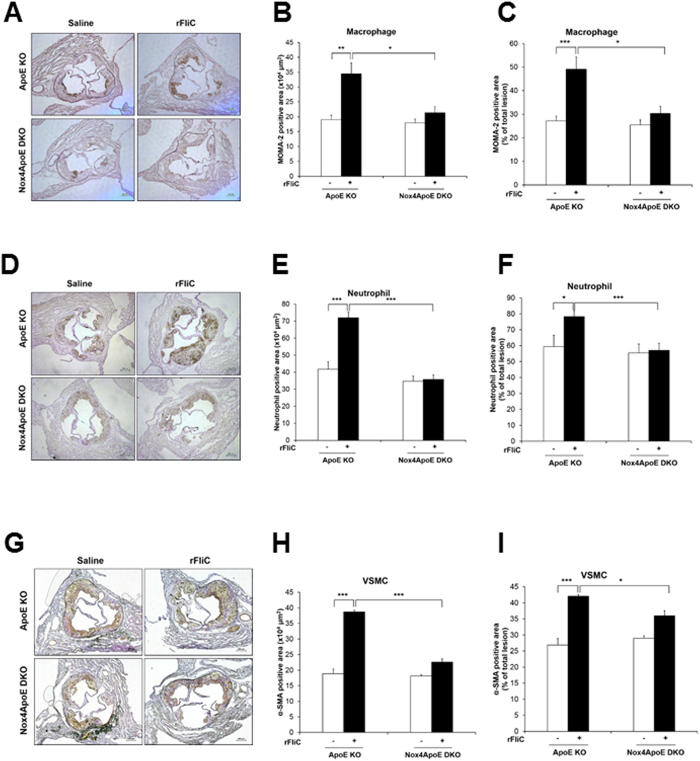
Analysis of atherosclerosis plaque composition in ApoE KO or Nox4ApoE dKO mice. (**A**) Sinus sections were obtained from ApoE KO or Nox4ApoE DKO mice fed high-fat diet for 12 weeks with or without rFliC injection. Each samples was stained with anti-monocyte/macrophage antibody (MOMA-2, AbD Serotec). (**B**) Quantification of MOMA-2 positive area in sinus sections from ApoE KO or Nox4ApoE DKO mice (N = 5 to 10, mean ± SEM, *p < 0.05, **p < 0.01). (**C**) Quantification of MOMA-2 positive area in total lesion area, N = 5 to 10, mean ± SEM, *p < 0.05, ***p < 0.001. (**D**) Sinus sections were stained with anti-neutrophil antibody (Abcam). (**E**) Quantification of neutrophil positive area in sinus sections from ApoE KO or Nox4ApoE DKO mice using image J (N = 5 to 10, mean ± SEM, ***p < 0.001). (**F**) Quantification of neutrophil positive area in total lesion area, N = 5 to 10, mean ± SEM, *p < 0.05, ***p < 0.001. (**G**) Sinus sections were stained with anti-smooth muscle actin (Dako). (**H**) Quantitative analysis of anti-smooth muscle actin positive area, N = 5 to 10, mean ± SEM, ***p < 0.001. (**I**) Quantification of smooth muscle actin positive area in total lesion area, N = 5 to 10, mean ± SEM, *p < 0.05, ***p < 0.001.

The flagellin-induced Nox4 activation stimulates the expression of various pro-inflammatory mediators, leading to the adhesion and trans-endothelial migration of monocytes which are the key events of atherosclerosis. To confirm the cellular events *in vivo*, we prepared a chronic atherosclerosis model in ApoE KO and Nox4ApoE DKO mice on a HFD for 12 weeks with or without rFliC. Atherosclerotic plaques in the mice were examined from the aortic arch to the abdominal aorta. Challenging ApoE KO mice with rFliC resulted in induction of atherosclerotic lesions in the aorta (Fig. 7A,B). However, injection of rFliC into the Nox4ApoE DKO mice failed to induce atherosclerotic plaque formation (Fig. 7A,B). To validate the effect of TLR5 on atherosclerosis, we injected rFliC or ΔrFliC, non-functional flagellin, into the ApoE mice with HFD. Injection of ApoE KO mice with rFliC resulted in induction of atherosclerotic lesions in the aorta, whereas ΔrFliC failed to induce atherosclerotic plaque formation (Fig. 7A,B). We further investigated characteristics of atherosclerotic plaques in sinus valve with hematoxylin/eosin (H&E) and Masson’s trichrome staining. Atherosclerotic plaques and necrotic core size in sinus valve of ApoE mice with rFliC injection were significantly increased compared to Nox4ApoE DKO mice (Fig. 7C–E). We examined infiltration of monocyte and neutrophil, accumulation of collagen and migration of vascular smooth muscle cells (VSMCs) in sinus valve of ApoE KO and Nox4ApoE DKO with HFD in the absence or presence of flagellin. Sinus valve of ApoE KO and Nox4ApoE DKO with HFD was stained with MOMA-2 as a macrophage marker and antibodies against neutrophils or smooth muscle actin (SMA) as a VSMA marker. Infiltration of macrophages and neutrophils, accumulation of collagen and migration of VSMCs into sinus valve in ApoE KO mice with rFliC was significantly increased. However, Nox4ApoE DKO mice was resistant to macrophage and neutrophil infiltration, collagen accumulation and VSMCs migration into sinus valve in response to rFliC injection (Figs 7F and 8). Moreover, ratio of macrophage, neutrophil, collagen and VSMCs content to total lesion area in sinus valve of Nox4ApoE KO mice were attenuated, compared to that of ApoE KO mice (Figs 7G and 8C,F,I). Thus, Nox4 deficiency abrogated HFD-induced atherosclerotic lesion formations as well as rFliC-mediated atherosclerosis including infiltration of monocyte and neutrophil, accumulation of collagen, and migration of VSMCs in ApoE KO mice indicating that Nox4 is an important mediator in the development of atherosclerosis.

## Discussion

It has been reported that bacterial infection is a high risk factor for atherosclerosis. Growing bodies of evidence indicate that periodontal disease caused by dental plaque microorganisms such as *Porphyromonas gingivalis* and *Actinobacillus actinomycetemcomitans* is related to atherosclerosis^6^,^30^. Infections of *Chlamydia pneumonia* and *Helicobacter pylori* have also been implicated in the pathogenesis of atherosclerosis. TLRs, as representative receptors for innate immune responses, play crucial roles in inflammatory diseases, including atherosclerosis^31^. It has been reported that the expression of TLR5 was up-regulated in human atheromatous tissue, indicating that vascular cells expressing TLR5 respond to flagellin during the development of carotid atheroma^10^,^11^. We first provided a molecular link between TLR5-mediated H_2_O_2_ generation and atherosclerosis. We demonstrated that TLR5-induced Nox4-dependent H_2_O_2_ generation is indispensable for the signaling events, including the production of pro-inflammatory cytokines and the expressions of surface adhesion molecules for atherogenesis (Figs 4, 5, 6A and 7). Moreover, we demonstrated that the Nox4-deficient ApoE KO mice exhibited significant protections against rFliC-mediated atherogenesis indicating that TLR5-dependent Nox4 activation is critical for the development of atherosclerosis.

Previously, we reported that Duox2 in the nasal airway epithelium plays an important role in TLR5-dependent inflammatory responses, including IL-8 production and the MUC5AC expression^14^. The nasal epithelium did not express the Nox4 isozyme, and the expression of Duox2 was hardly detected in the aortic endothelial cells (Supplementary Figure S8). These results suggested that the regulation of Nox isozymes by TLR5 was displayed in a cell type-specific or tissue-specific manner. The activity of the Duox2 isozyme in the nasal airway epithelium was regulated by intracellular calcium mobilization which was regulated by flagellin-TLR5. On the other hand, the association of TLR5 with Nox4 can directly regulate Nox4 activity. In this study, we showed that flagellin stimulated H_2_O_2_ generation through Nox4 activation (Fig. 2). However, detailed mechanism underlying the interaction between TLR5 and Nox4 that leads to H_2_O_2_ generation remains to be clarified. It has been reported that TLR5-mediated inflammation is regulated by the MyD88-dependent pathway. In our data, interestingly, MyD88 is not necessary for H_2_O_2_ generation in response to flagellin in MAECs (Fig. 3E), indicating that TLR5 stimulates Nox4 activity through the non-canonical MyD88-independent pathway. Although MyD88 was not involved in the TLR5-Nox4 axis, TLR5 induced the nuclear localization of RelA/p65 as a subunit of NFkB. Moreover, knockdown of Nox4 or TLR5 expression in HAECs failed to induce the nuclear localization of RelA/p65 (Fig. 3F,G). However, how the non-canonical TLR5-Nox4 axis is linked to the nuclear localization of NF-kB remains to be elucidated.

Several lines of evidence suggest that Nox isozymes are associated with the inflammation of vascular cells that lead to atherosclerosis^32^,^33^. Although the expression of Nox2 has been reported in phagocytic cells, the atherogenic condition, such as the challenging of oxidized LDL and turbulent flow, stimulated the induction of the Nox2 expression in endothelial cells. The endothelium-specific Nox2 overexpression in ApoE KO mice resulted in increased ROS generation and macrophage infiltration into the intima^34^. Moreover, Nox2ApoE DKO mice and p47phoxApoE DKO mice with HFDs for 12 weeks experienced reduced atherosclerotic lesions in the aortic arches and descending aortas compared with ApoE KO mice, indicating that the activation of Nox2 contributes to the early events of atherogenesis. However, the function of Nox1 in atherogenesis is controversial. The effect of Nox1-deficient ApoE KO with HFDs on the development of atherosclerotic lesions was small^35^. The Nox4 isozyme is highly expressed in endothelial cells. Moreover, the expression of Nox4 is induced by the stimulation of oxidized LDL, oscillatory shear stress, and oxidized-1-palmitoyl-2-arachidonoyl-sn-glycero-3-phosphorylcholine (oxPAPC)^36^. Even if Nox4 was a major isoform and the expression was regulated in atherosclerotic lesions, the function of Nox4 in atherogenesis is far from clear. Here, we clearly demonstrated that Nox4 plays an important role in the early events of atherogenesis in Nox4ApoE DKO mice with HFDs in the absence or presence of rFliC stimulation (Fig. 7A,B). Atherosclerotic lesions in the descending aortas of ApoE KO mice with HFDs diminished in Nox4ApoE DKO mice.

**Figure 9:**
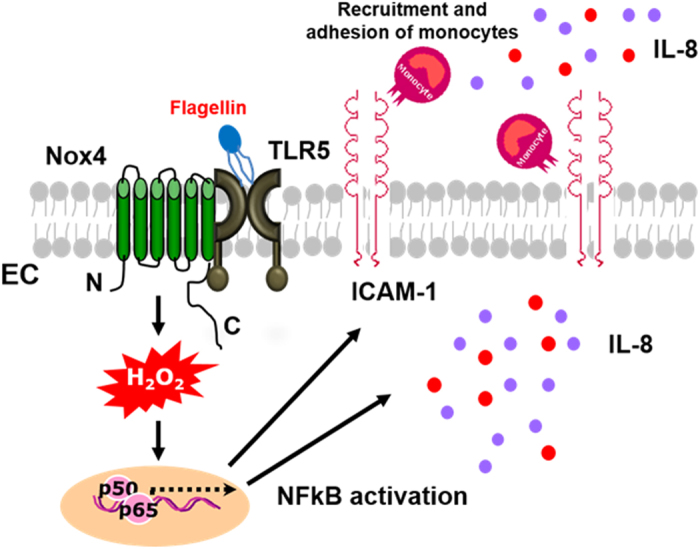
Proposed model of TLR5-Nox4 complex function in atherosclerosis. Interaction of TLR5 with Nox4 plays an essential role in atherosclerosis. Ligation of TLR5 with flagellin stimulated Nox4 to generate H_2_O_2_ which in turn promoted the secretion of pro-inflammatory cytokines including IL-8 and IL-6, as well as the expression of ICAM-1 in endothelial cells. The expressions of pro-inflammatory mediators induced binding and transmigration of monocytes, leading to the development of atherosclerotic lesions in the aortas. It demonstrated that activation of TLR5-Nox4 cascade contributed to the early events of atherogenesis.

Moreover, the expressions of pro-inflammatory mediators, including ICAM-1, VCAM-1, and IL-8 in endothelial cells of ApoE KO were downregulated in those of the Nox4ApoE DKO mice. Injections of rFliC into the ApoE KO mice resulted in increased expressions of pro-inflammatory mediators, leading to the development of atherosclerotic lesions in the aortas (Fig. 6). However, knockout of Nox4 in ApoE KO mice revealed the attenuated expressions of pro-inflammatory mediators and atherosclerotic lesions. MCP-1 (monocyte chemoattractant peptide-1) plays an important role in atherosclerosis^22^. Therefore, we further examined production of MCP-1 in HAEC in response to flagellin stimulation. Quantity and expression pattern of MCP-1 were similar between control siRNA- and Nox4 siRNA-transfected HAECs (Supplementary Figure S9A). However, MCP-1 production in MAECs from wild type and Nox4 KO mice showed different pattern. Stimulation of Nox4 KO MAECs with flagellin resulted in attenuated MCP-1 production, compared to MAECs from wild type (Supplementary Figure S9B). Reduced MCP-1 production in Nox4 KO MAECs contributed to macrophage infiltration in sinus valve of Nox4ApoE DKO with HFD (Fig. 8A–C). Taken together, these results indicate that the activation of Nox4 contributed to the early events of atherogenesis (Fig. 9).

In conclusion, H_2_O_2_ generation through Nox4 activation plays a significant role in mediating flagellin-induced atherosclerosis, and we first verified a link between TLR5 and atherosclerosis. Targeting the TLR5-Nox4 cascade in endothelial cells could be a possible therapeutic strategy for atherosclerosis.

## Methods

### Mice

Nox1 KO mice and MyD88 KO mice were purchased from Jackson laboratory. Generation of Nox4 KO mice were described in a previous report^17^. Nox4 KO mice were crossed with ApoE KO mice to generate Nox4ApoE double knockout mice. The study was reviewed and approved by the Institutional Animal Care and Use Committee (IACUC) of Center for Laboratory Animal Sciences, Ewha Industry-University Cooperation Foundation, Ewha Womans University. Experimental methods were carried out in accordance with the approved ethic guidelines in Ewha Industry-University Cooperation Foundation, Ewha Womans University.

### Isolation of murine aortic endothelial cells

Murine aortic endothelial cells (MAECs) were isolated by described previously^37^. The purity of the MAEC preparations was confirmed with DiI-ac-LDL (Biomedical Technologies) uptake and PECAM-1 staining.

### Measurement of intracellular H_2_O_2_

Measurement of intracellular H_2_O_2_ using DCF-DA and Peroxy Orange-1 (PO-1) were performed as previously described^38^.

### Small interfering RNA (siRNA)

Transfection of siRNA into HAECs was performed with lipofectamine RNAi MAX (Invitrogen) according to the manufacturer’s instructions. ON-TARGET plus SMARTpool human Nox4 (Dharmacon) was used for knockdown of Nox4 in HAECs. ON-TARGET plus SMARTpool human TLR5 (Dharmacon) was used for knockdown of TLR5 in HAECs. For control, ON-TARGET plus non-targeting siRNA (Dharmacon) was used.

### Quantitative real time PCR analysis

Quantitative real time PCR was performed with KAPA probe fast universial 2x qPCR master mix (KAPA Biosystems) for analyzing NADPH oxidase isozymes’ expression level using Taqman gene expression assay (Applied Biosystems). GAPDH was a house-keeping control. Other gene expression was quantified with KAPA SYBR® fast qPCR kit (KAPA Biosystems) using the following specific primers: human ICAM-1, 5′-AGCCCAAGTTGTTGGGCATA-3′ and 5′-AGTCCAGTACACGGTGAGGA-3′; human IL-8, 5′-TCAGAGACAGCAGAGCACAC-3′ and 5′-GGCAAAACTGCACCTTCACA-3′; human VCAM-1, 5′-TGGGCTGTGAATCCCCATCT-3′ and 5′-GGGTCAGCGTGGAATTGGTC-3′; human Klf2, 5′-AGACCTACACCAAGAGTTCGCATC-3′ and 5′-CATGTGCCGTTTCATGTGCAGC-3′; mouse ICAM-1, 5′-AGGTGGTTCTTCTGAGCGGC-3′ and 5′-AAACAGGAACTTTCCCGCCA-3′; mouse VCAM-1, 5′-TCTTGGGAGCCTCAACGGTA-3′ and 5′-CAAGTGAGGGCCATGGAGTC-3′; mouse MIP-2, 5′-GCCCAGACAGAAGTCATAGC-3′ and 5′-CTCCTCCTTTCCAGGTCAGTT-3′; mouse KC, 5′-TGGCTGGGATTCACCTCAAG-3′ and 5′-TCTCCGTTACTTGGGGACAC-3′; human & mouse 18S, 5′-AGGAATTGACGGAAGGGCACCA-3′ and 5′-GTGCAGCCCCGGACATCTAAG-3′. 18S was a house-keeping control.

### Measurement of ICAM-1 protein expression level

HAECs and MAECs were stained with a Phycoerythrin (PE) conjugated anti-human CD54 (ICAM-1) (eBioscience). Control was stained using a mouse IgG1 isotype antibody conjugated with PE (eBioscience). HAECs were incubated with or without flagellin (invivogen, cat# tlrl-stfla, 100 ng/ml) for 16 hrs. Cells were removed from the culture flask by trypsinization. Subsequently, the cells were incubated with the respective monoclonal antibody for 1 hr at 4 °C. Cells were washed with PBS, resuspended in PBS and subjected to flow cytometry in a fluorescence-activated cell sorter (FACScan, Becton and Dickinson, Mountain View, CA).

### Determination of IL-8 protein secretion level

HAECs and MAECs were incubated with or without flagellin (100 ng/ml) for 12 hrs, and then the medium was collected. IL-8 protein levels were determined by ELISA assay kit according to the manufacturer’s instruction (R&D systems).

### Monocyte adhesion assay

HAECs were treated in the absence or presence of flagellin (100 ng/ml) for 6 hrs, U937 cells were stained with 5 μg/ml of BCECF-AM (Molecular Probes) for 30 min. U937 cells (5 × 10^5^ cells) were added and allowed to adhere to HAECs. After 30 min, cells were washed twice to remove any non-adherent U937 cells, and adherent U937 cells were fixed with 4% paraformaldehyde. After washing with PBS three times more, the samples were visualized with the fluorescent microscope (Axiovert 200).

### Monocyte adhesion in shear stress condition

HAECs (8 × 10^5^ cells) were cultured on 100 mm dishes and transfected with control siRNA or Nox4 siRNA for 24 hrs. Confluent HAECs were exposed to steady LS (15 dyn/cm^2^), OS (±5 dyn/cm^2^) or static conditions for 24 hr using a cone-and-plate shear device with or without flagellin (100 ng/ml). After shear stress, U937 cells (5 × 10^5^ cells) were added and allowed to adhere to HAECs for 30 min. Cells were washed twice to remove any non-adherent U937 cells, and adherent U937 cells were fixed with 4% paraformaldehyde and stained with BCECF-AM (5 μg/ml). After washing with PBS three times more, the samples were visualized with the fluorescent microscope (Axiovert 200).

### Monocyte migration assay

Migration assays were performed in a Transwell system (Transwell, Costar; Cambridge, MA). Transwells (polycarbonate membranes with 5 μm pore sizes) in 24-well plates were used. For transendothelial migration, HAECs were cultured on the lower compartment. HAEC monolayers were activated with 100 ng/ml flagellin for 4 hrs, and U937 monocytes (5 × 10^5^ cells) were added to the upper compartment. After 2 hrs of incubation, the membrane was recovered and fixed in methanol and stained by H&E. Images were captured using Nickon Eclipse 80i microscope (Nickon).

### rFliC purification and elimination of endotoxin

*Salmonella enteritidis* FliC flagellin gene (GenBank accession: No. M84980) was obtained by PCR. Wild type FliC (AA21–505) and deletion mutant of FliC (AA119–505) were cloned in the pET15b vector to generate His epitope-tagged recombinant protein. Expression of the fusion proteins was induced with 0.4 mM IPTG at 30 °C for 3 hrs, and the cells were lysed by sonication. After centrifugation for 30 min at 14,000 rpm, the resulting supernatant was incubated with Ni^+^-NTA resin (Qiagen) at 4 °C for 1 hr. The recombinant FliC protein and mutant ΔFliC protein were eluted from the resin with elution buffer containing 50 mM phosphate (pH 7.4), 20 mM NaCl, 100 mM imidazole and protease inhibitors. Recombinant FliC and mutant ΔFliC protein were passed through an EndoTrap Red endotoxin removal column (Hyglos) to remove potential endotoxin according to the manufacturer’s instructions. Endotoxin level was measured using kinetic-QCL limulus amebocyte lysate (LAL) assay (Lonza). LAL assays showed an endotoxin content of <0.1 EU/ml in the purified rFliC and mutant rFliC (ΔrFliC). Standard flagellin from *S.typhimurium* was purchased from Invivogen (cat# tlrl-stfla). Flagellin contained 0.1–1EU endotoxin in this experiment.

### Intimal RNA preparation from mouse thoracic aorta

Intimal RNA preparation from mouse thoracic aorta was prepared as previously described^27^. Thoracic aorta was isolated and carefully cleaned of peri-adventitial fat. The aorta lumen was quickly flushed (few seconds) with 200 μl of QIAzol lysis reagent (QIAGEN) using a 25-gauge syringe in a microfuge tube. The eluents was then used for intimal RNA isolation using a miRNeasy mini kit (QIAGEN) according to the manufacturer’s instructions.

### 4-HNE staining

ApoE KO and Nox4ApoE DKO mice were received intravenous injection of saline or rFliC (25 μg/mouse). After 2 hrs, the thoracic aorta were isolated and were embedded in Frozen Section Compound (Leica), frozen on dry ice. Five cryosections (each 10 μm thick) were obtained in each animal. The samples were incubated with 4-hydroxynonenal antibody (Abcam) at 4 °C for overnight. An Alexa Fluor® 594-conjugated goat anti-mouse IgG was used as the second antibody.

### Atherosclerotic lesion analysis

ApoE KO and Nox4ApoE DKO mice (12-wk) were received intravenous injection of saline, rFliC, or ΔrFliC (25 μg/mouse) every week for 3 months with the Paigen’s high-fat diet (HFD; Research Diet) containing 1.25% cholesterol, 15% fat, and 0.5% cholic acid. After 3 month, mouse aorta was isolated from the proximal ascending aorta to abdominal aorta, and adventitial fat was removed. Isolated aorta was stained with Oil-red-O for 2 hrs, pinned flat onto black silicon plates, and photographed at a fixed magnification. The photographs were digitized, and total aortic areas and lesion areas were calculated by using Image J software. The results were reported as a percentage of the total aortic area that contained lesions.

### Histological analysis

ApoE KO or Nox4ApoE DKO mice fed high-fat diet for 12 weeks with or without rFliC injection were euthanized and perfused with PBS via the left ventricle after severing the inferior vena cava. Hearts were harvested, fixed for 24 h with 10% formalin. The tissue embedded in Frozen Section Compound (Leica), frozen frozen on dry ice and 10-μm cross sections were prepared. Sinus valve were stained with H&E or Masson’s trichrome stain kit (Sigma). Samples were imaged using a Nickon Eclipse 80i microscope (Nickon) and analyzed using Image J software.

### Immunohistochemistry

Frozen sections were incubated with primary antibody overnight at 4 °C. The primary antibodies used were MOMA-2 (1:25 dilution; AbD Serotec), α-smooth muscle actin (1:200 dilution; Dako), and neutrophil (1:50 dilution; Abcam). As a negative control, species- and isotype-matched IgG were used in place of the primary antibody. Counterstaining was with hematoxylin. Slides were imaged using a Nickon Eclipse 80i microscope (Nickon) and analyzed using Image J software.

### Statistical analysis

Data are given as mean ± SD or mean ± SEM. Pairwise comparisons were performed using student T-tests. Multiple comparisons of means were calculated using one-way analysis of variance (ANOVA) test (GraphPad software). Differences between groups were considered significant at P values below 0.05.

## Additional Information

**How to cite this article**: Kim, J. *et al.* Flagellin-induced NADPH oxidase 4 activation is involved in atherosclerosis. *Sci. Rep.* **6**, 25437; doi: 10.1038/srep25437 (2016).

## Supplementary Material

Supplementary Information
