# Supplementary material for: Flagellin-induced NADPH oxidase 4 activation is involved in atherosclerosis
Source: Sci Rep. 2016 May 5;6:25437. doi: 10.1038/srep25437 (PMC4857127; doi:10.1038/srep25437)
Supplement: Supplementary Information [file srep25437-s1.pdf]

Supplementary Information

Flagellin-induced NADPH oxidase 4 activation is involved in atherosclerosis

Jinoh Kim[1], Misun Seo[1], Su Kyung Kim[1], Yun Soo Bae[1*]

[1]Department of Life Science, Ewha Womans University, Seoul, Korea

Correspondence: *Yun Soo Bae, Department of Life Science, Ewha Womans University, Daehyun-Dong, Seodaemoon-Gu, Seoul 120-750, Korea, Phone: 82-2-3277-2729, fax: 82-2-3277-3760, e-mail: baeys@ewha.ac.kr

Kim et al. Supplementary Figure S1

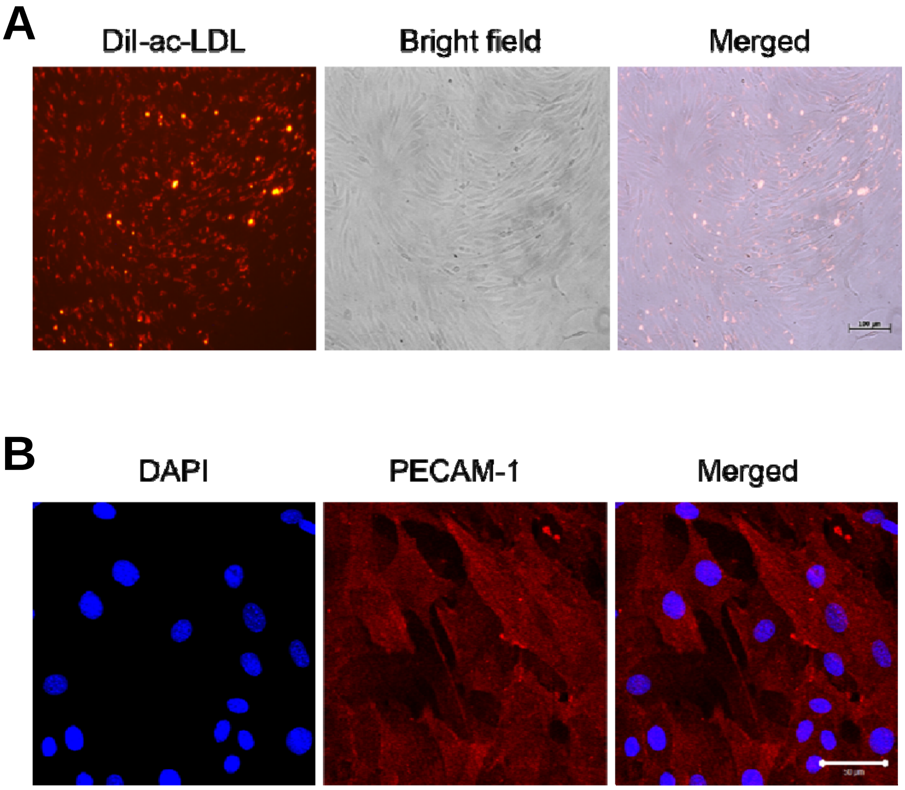

Supplementary Figure S1. Isolation of mouse aortic endothelial cells (MAECs). (A) MAECs were incubated with Dil-Ac-LDL (10 μg/mL) for 4 h, and images were taken by fluorescence microscopy, scale bar : 100 μm. (B) MAECs were immunostained using the endothelial markers, PECAM-1 and images were taken by confocal microscopy, scale bar : 50 μm.

Kim et al. Supplementary Figure S2

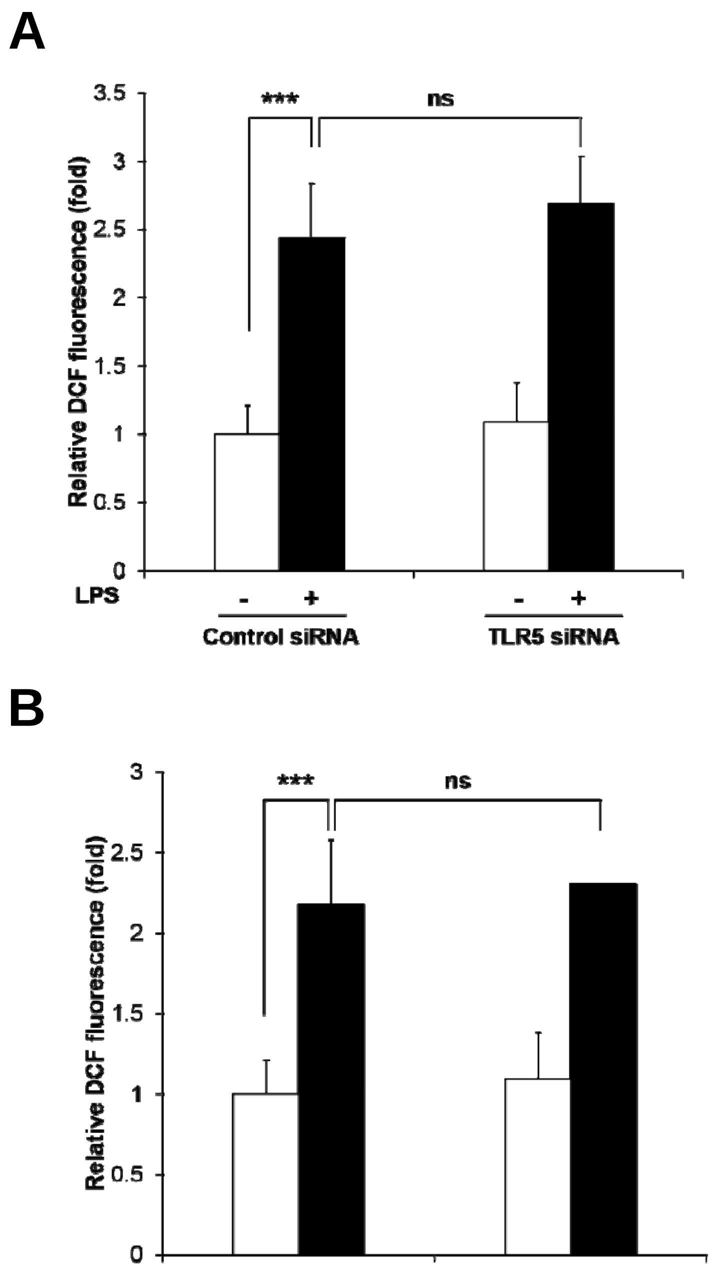

Supplementary Figure S2. Effect of TLR5 on LPS- or PAM3CSK4-dependent ROS generation. HAECs transfected control or TLR5 siRNA were stimulated for 10 min with LPS (100 ng/ml), and Pam_3CSK_4 (1 µg/ml). H_2O_2 was monitored by a confocal microscopic analysis of DCF fluorescence (N = 3, mean ± SD, ***$p<0.001$).

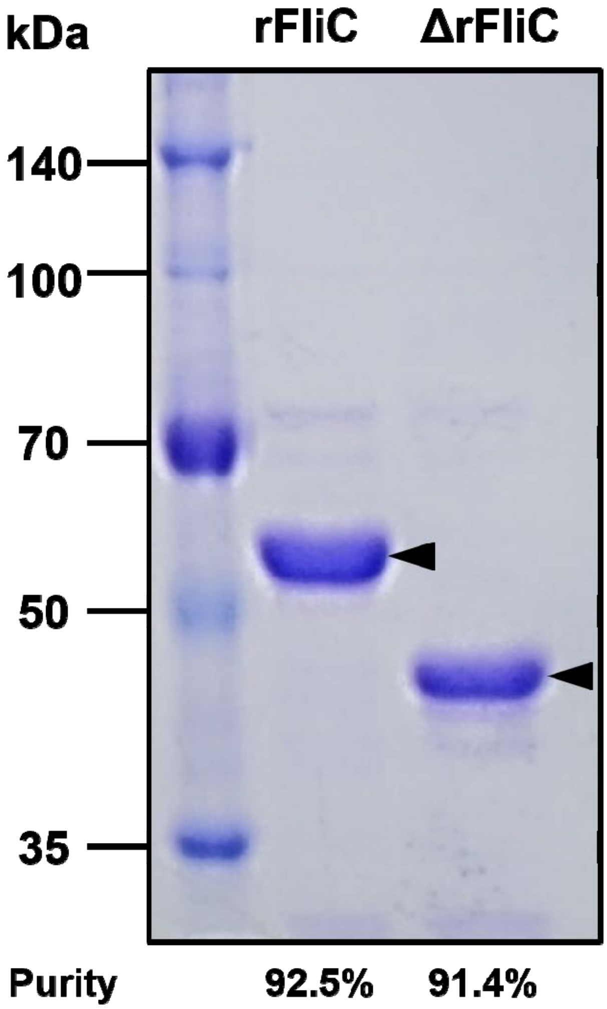

Supplementary Figure S3. Purity of rFliC and ΔrFliC. Purified recombinant FliC protein (rFliC) and mutant FliC protein (ΔrFliC) were separated by 8% SDS-PAGE gel and visualized using Coomassie blue staining.

Kim et al. Supplementary Figure S4

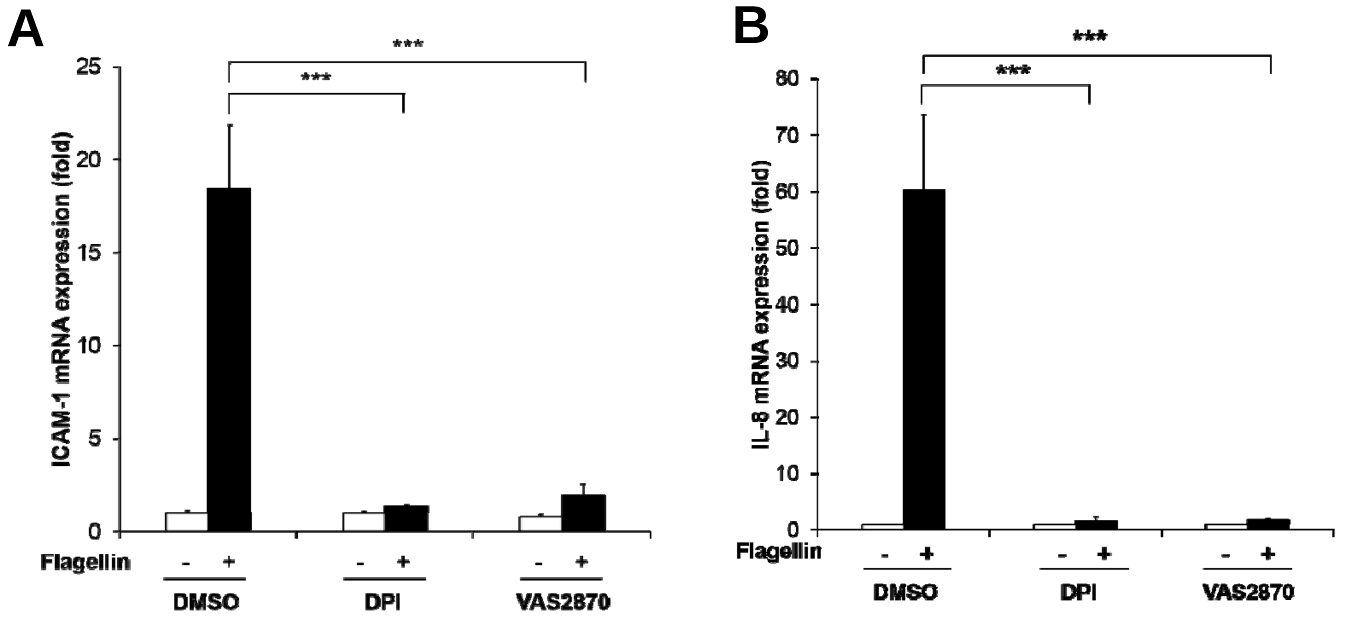

Supplementary Figure S4. Effect of Nox inhibitor on production of ICAM-1 and IL-8 in HAECs. HAECs were serum starved for 16hrs and incubated with DMSO, DPI (20 µM), or VAS2870 (5 µM) for 30min, then stimulated with flagellin (100 ng/ml) for 2 hrs. (A) Quantification of ICAM-1 mRNA expression (N = 3, mean ± SD, ***$p<0.005$). (B) Quantification of IL-8 mRNA expression (N = 3, mean ± SD, ***$p<0.001$).

Kim et al. Supplementary Figure S5

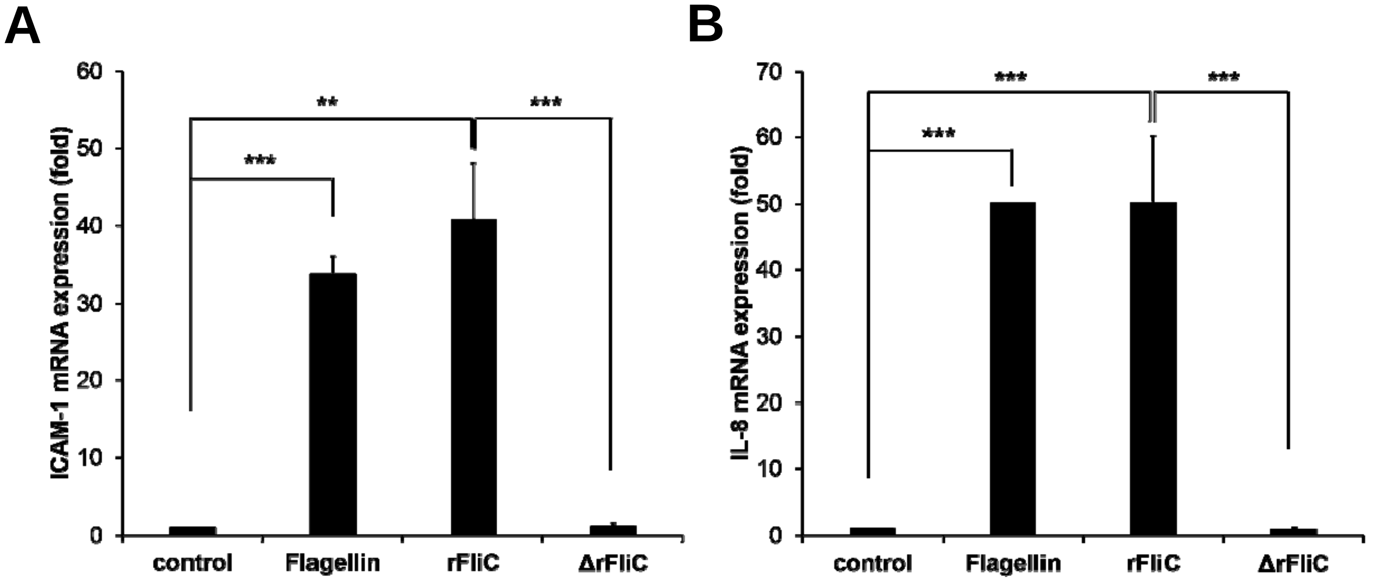

Supplementary Figure S5. rFliC mutant does not produce ICAM-1 and IL-8 in HAECs. HAECs were stimulated with flagellin (100 ng/ml), rFliC (100 ng/ml), or ΔrFliC (100 ng/ml). (A) Quantification of ICAM-1 mRNA expression (N = 3, mean ± SD, ***p<0.001, **p<0.01). (B) Quantification of IL-8 mRNA expression (N = 3, mean ± SD, ***p<0.001).

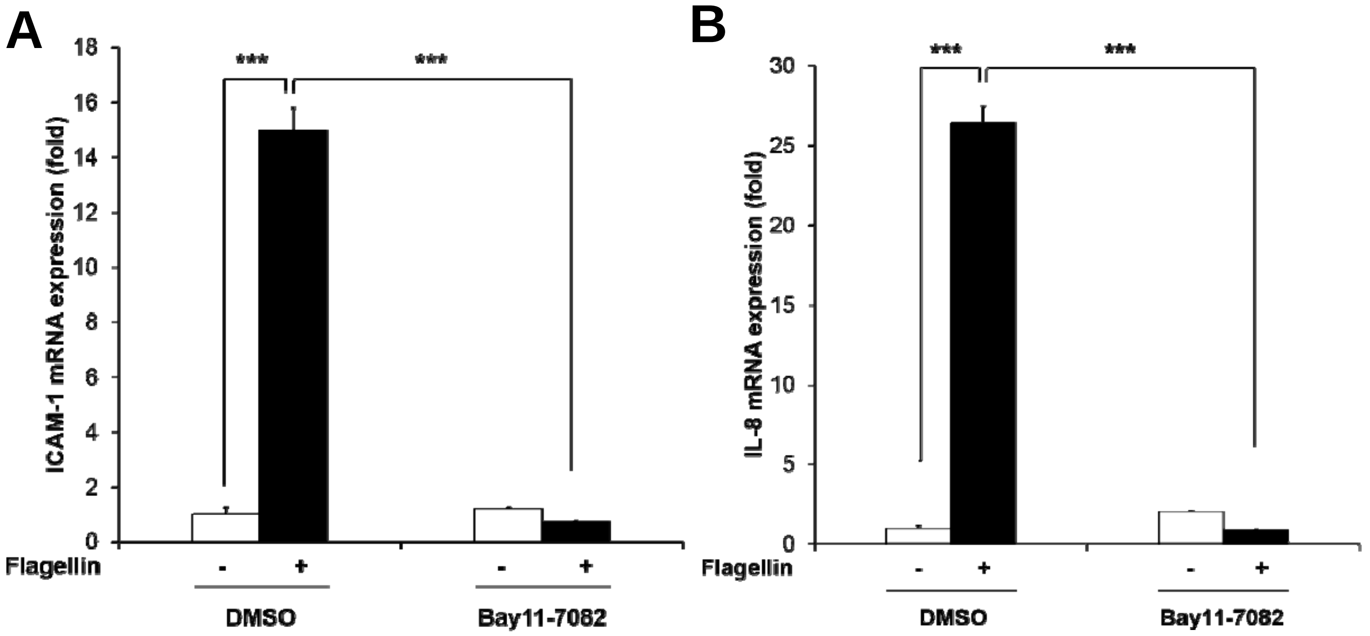

Supplementary Figure S6. Inhibition of NF-kB signaling decrease the flagellin-induced ICAM-1 and IL-8 production in HAECs. HAECs were incubate with DMSO or Bay11-7082 (10 μM) for 3 hrs, then stimulated with flagellin (100 ng/ml) for 2 hrs. (A) Quantification of ICAM-1 mRNA expression (N = 3, mean ± SD, ***$p<0.001$). (B) Quantification of IL-8 mRNA expression (N = 3, mean ± SD, ***$p<0.001$).

Kim et al. Supplementary Figure S7

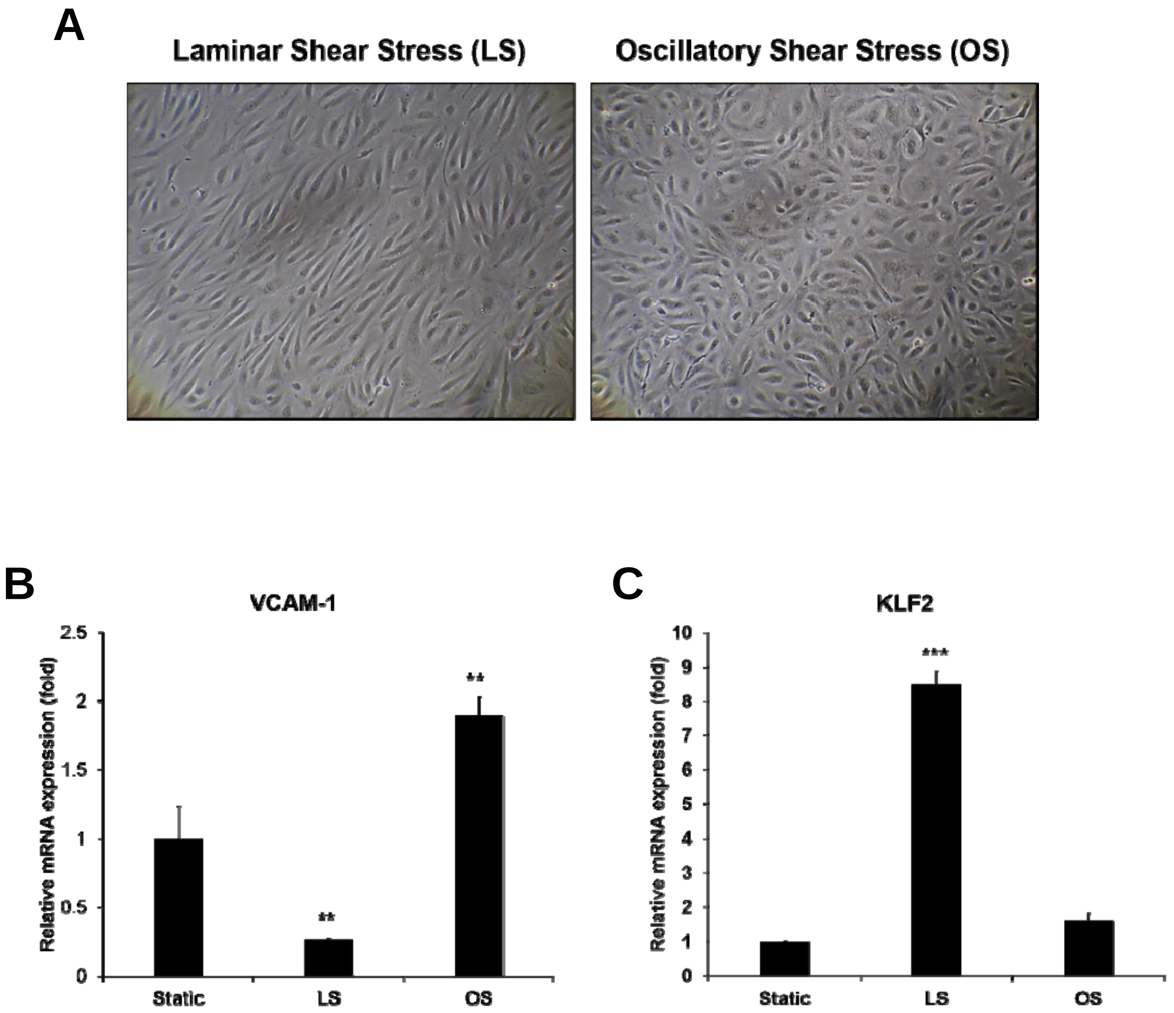

Supplementary Figure S7. Morphological changes and gene expression in flow condition. (A) Morphology of HAECs after shear stress. Confluent HAECs were exposed to laminar shear stress (15 dyn/cm^2) or oscillatory shear stress (±5 dyn/cm^2) for 24 hrs using the cone-and-plate apparatus (B-C). Confirmation of shear stress condition. (B) VCAM-1 mRNA expression, mean ± SD, **$p<0.01$ vs static. (C) Klf2 mRNA expression, mean ± SD, ***$p<0.001$ vs static.

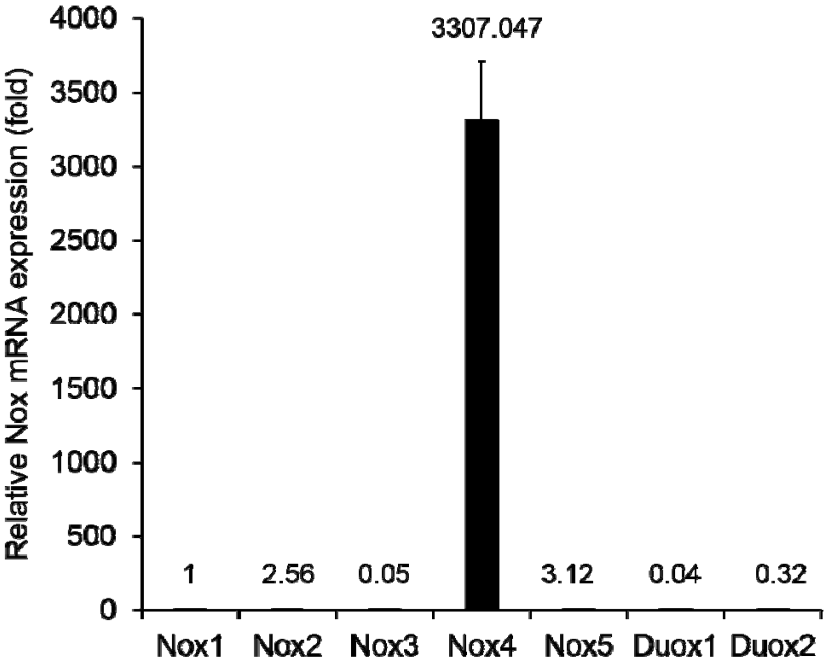

Supplementary Figure S8. Nox4 is the major isozyme in human aortic endothelial cells. Quantification of Nox isozyme expression level in human aortic endothelial cells. Total RNA was isolated from HAECs, reverse transcribed, and quantified by real-time PCR with Nox1-Nox5, Duox1, 2 primers. The results are normalized by GAPDH. (N = 3, data shown as mean ± SD)

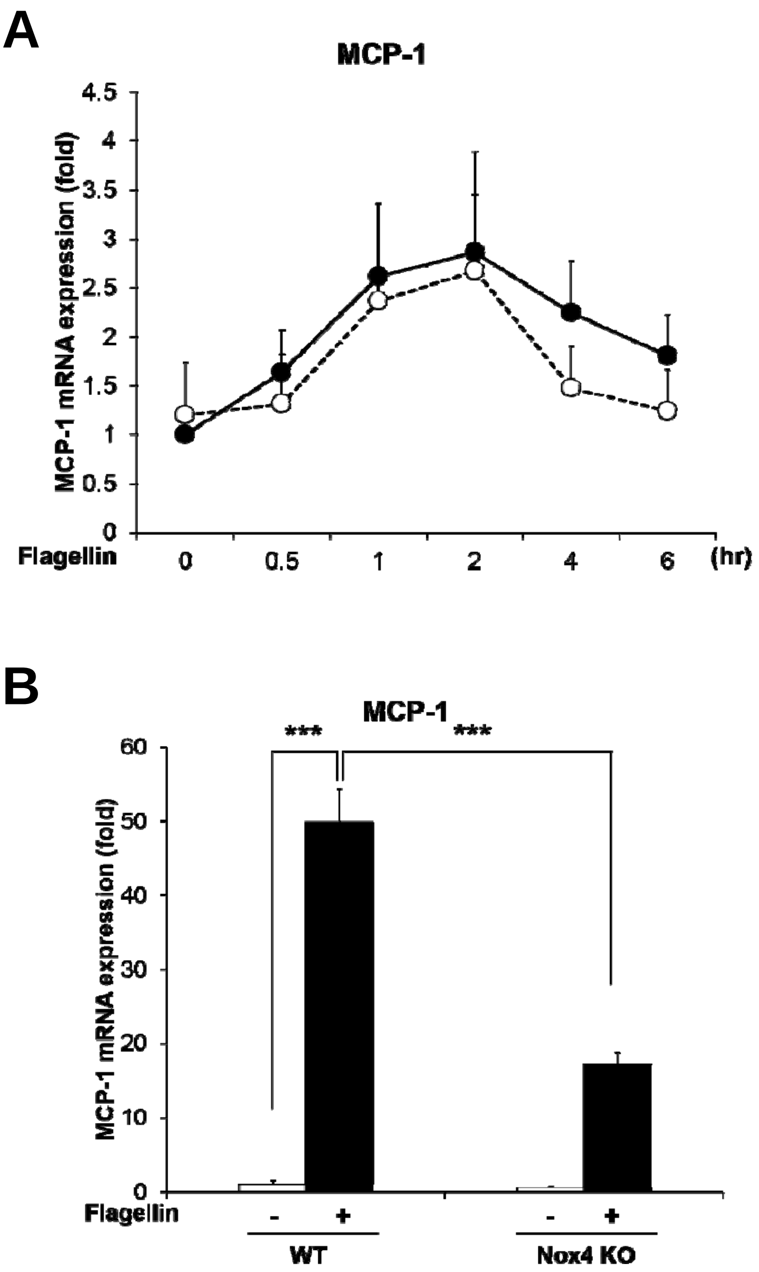

Supplementary Figure S9. Effect of Nox4 on MCP-1 production. (A) Quantification of flagellin-dependent MCP-1 mRNA expression in HAECs transfected control or Nox4 siRNA (N = 3, mean ± SD). (B) Quantification of flagellin-induced MCP-1 production in MAECs from wild type or Nox4 KO mice (N = 3, mean ± SD, ***$p<0.001$).
